# Transactions of the Medical Association of Southern Central New York, at the Annual Meeting Held at Owego, June, 1852

**Published:** 1852-10

**Authors:** 


ART- IV.—*Transactions of the Medical Association of Southern Central New York, at the Annual Meeting held at Owego,* June, 1852, —p. p. 94, 8vo.

These transactions open with the annual address of the President, Dr. James H. Jerome, who takes a passing notice of the principal systems of medicine which have successively prevailed. We find nothing in it worth copying.

The second paper is a report, by Dr. Green, on the vital statistics of Cortland county. The report closes with the following practical suggestions:

We are fully impressed with the conviction that country physicians, especially, could not better discharge their obligations to community, as guardians of the public health, than by an earnest recommendation of the simple use of *woolen flannel* next to the skin, and to a majority of persons during the whole year. We fully believe that it would, if generally adopted, very greatly lessen the frequency and severity of cases of congestion and inflammation of the digestive organs, and of the respiratory apparatus ; and especially that the two diseases which often claim the highest number of victims in our county, viz: dysenteria and pulmo-tuberculosis, would be astonishingly diminished. If the abdomens of children were protected, during the summer months, with flannel, it would serve as a most efficient prophylactic against the bowel affections, to which they are so obnoxious. The common catarrhal affections which are often as prevalent in summer as in winter, might, in a great measure, be prevented by the use of flannel ; or when they do occur, their cure might be rapidly promoted by a resort to the flannel wrapper, or by the simple expedient of lining the back of the shirt with a strip of flannel, thus protecting the spine from sudden atmospheric vicissitudes.

The third article is a very condensed and brief report on the vital statistics of Owego, by Dr. L. H. Allen; which is succeeded by a report, by Dr. F. Hyde, on the surgery of Cortland county. The following is the history of an accident which, for the want of judicious early treatment, rarely has as favorable a termination as in this instance:

*Incised Wound Opening the Knee-joint.*—The following case occurred under the observation of Dr. L. S. Keene, of McGrawville, a subjoined sketch of which he has furnished the committee. A lad of eleven years fell from a fence, striking his knee on the edge of a sharp scythe, cutting open and disarticulating the knee, leaving only the ham-strings, large nerves, and blood-vessels undivided. A small piece of the head of the fibula was shaved off, otherwise the wound was directly through the joint, exposing the entire articulation.

*Treatment.*—The limb was placed in the straight position, one of the articular arteries tied, the edges of the wound brought together, maintained by sutures and adhesive plasters, on which were applied a rattan and splints. Anti-inflammatory measures were pursued.—Two-thirds of the wound united by first intention; the remainder healed kindly, after which the parts were subjected to slight motion daily. In two months from the receipt of the injury, he could walk by drawing the limb after him. At the end of five months he regained the power of flexion and extension sufficiently to walk quite well. At this time there was considerable effusion in the joint, but it has been gradually absorbed.

The surgical report of Tioga county makes the next article, which begins with the following case of *cancrum oris:*

H. T., girl six years old, of scrofulous habit, has had occasional obstinate swellings about face and neck, since having the measles. Now convalescing from billious remitting fever. Oct. 23, 1851. Cried with tooth-ache, fever gone, tongue clean, some desire for food, pulse 120, soft. Oct. 24. There is swelling of right cheek, which is red and painful; cold applications not tolerated; warm poultice of hops and vinegar applied, with considerable abatement of swelling and alleviation of pain.

25th. Much the same; appetite improving. 26th. Discoloration upon inner side of cheek, and gums ash color; tumefaction remains same. Applied strong solution Nit. Silver. 27th. Grangrene spreading upon inner side of cheek; teeth of that side loose; fœtid breath; tongue clean and moist; skin soft; appetite good; general appearances all good; mortification approaching the surface externally, showing itself in dark line, to the right and a little below the angle of the mouth—appearing like a bruise. Administer Quinine and wine liberally; kreosote; poultice of yeast and charcoal to cheek, changed frequently. Drs. Hoyt and Kiff called in consultation this afternoon; concur entirely in diagnosis and treatment.

28th. Mortification gradually extending; same treatment continued.

29th. Drs. Everett, Cole, and Woodworth called—same treatment continued, in accordance with the advice of consulting physicians; mortification still extending.—It has reached the size of a dollar upon external surface—constitutional sympathy very considerable; appetite continued good—in short, the mortification continued to spread, the typhoid symptoms proportionably increased in severity until the 5th of November, when the patient died.

The next article consists of a report on surgery, by Dr. Burr, for the county of Broome. Of *Cancer*, the committee say:

One case of cancer, or encephaloid tumor, of the breas has occurred, or rather has terminated fatally within the year. Two operations for the removal of the diseased portion have been performed, but with no permanent good effect. The mother of this patient died a few years since with a simllar disease. Another member of the family is now said to be suffering from the same affection, in its incipient stage.

One case of cancer reported by Dr. French, has terminated fatally. Its seat was in the parotid gland.

*Spermatorrhœa.*—Among these cases *three* have been treated after Lallemand's method. In two cases great amendment has taken place. In the third no perceptible amendment. Others have been treated in the usual way: by tonics and moral admonitions.

Two cases of Phlegmonous inflammation, and a case of Staphyloma, consequent upon purulent ophthalmia, are then given, and after these we have the following instructive case of imperforate anus, reported by Dr. Squire:

In November last I attended Mrs. Hemmenway in confinement. The mother got well, but the child died at the end of the fourth day, having had no evacuation of meconium, on account of imperforate anus. Perhaps the only instructive and remarkable circumstance connected with this case, is the fact that this malformation was not discovered till the post mortem examination was made. It is true that I was led to suspect this difficulty early, but my suspicions were all removed by the mother's statement that there was an external opening to the bowels, for she had introduced some soap, for the purpose of inducing an evacuation. The next day I made an examination myself and administered an injection, which came away immediately, but nothing followed. Afterwards I gave mild cathartics, and finally aloes, and the child died of course.

Externally, the place where the anus should have been, was perfectly evident to the eye, and did not differ in appearance from the natural anus where its sphinctor is firmly contracted. When the nates were separated, and the integuments put upon a firm stretch, a slight fossa or sulucs appeared where the natural opening should have been, and the complete occlusion was readily perceived. The color and appearance of this small fossa, were not the same as of genuine integument, but looked more as I should imagine the parts would look, if the natural anus had been obliterated by a cicatrix, resulting from a burn.

The bones of the pelvis having been separated at the pubis, and the condition of the internal parts examined, the rectum was found to terminate by a perfect cul de sac, in the hollow of the sacrum, the space of an inch or so intervening between the closed gut and the closed sphincter. A sheath of areolar tissue which invested the rectum at its unnatural extremity, was condensed into a small fibrous cord or ligament, which was firmly attached to the perineum, where the anus should have been. No other deformity existed. I doubt whether an operation in such a case as this would prove satisfactory. Especially would I object to any attempt to form an artificial anus in the groin or lumber region, as some authors advise, when the operation in the perineum fails. In such unfortunate cases, it is the surgeons duty to desist.

Dr. Jerome's report of a case of poisoning with oxalic acid, forms the next article. As the cases of poisoning by this article have not yet been much multiplied, we quote this, which is thus stated :

In March last, an Irish girl in the family of Mr. ——, of Trumansburg, through mistake, supposing it to be epsom salts, took a table spoonful of oxalic acid. On learning which, the gentleman with whom she lived sent immediately for a physician. Dr. Clark and myself were called. I saw her some twenty minutes from the time of her taking the acid, at which time she was retching powerfully, with frequent eructations and much anxiety of countenance. She complained also of a burning sensation in the region of the fauces and throat.

We immediately gave her largely of ipecac and sulphate of zinc, and as much warm water as she could be induced to drink, both of which were repeated at intervals of but a few minutes. We also irritated the fauces with the feather of a quill, and in about eight minutes vomiting was induced and the stomach thoroughly emptied of its contents. The water and ipecac were repeated until the stomach was evacuated three or four times. Calcined magnesia was freely used during the time. The retching and vomiting ceased in about an hour. The other symptoms subsided, and the patient convalesced in three or four days, leaving no unpleasant traces of the poison, except a slight inflammation of the mucous membrane lining the mouth and stomach.

